# Performance of artificial intelligence in the collection of patient history in general practice

**DOI:** 10.1016/j.aprim.2026.103529

**Published:** 2026-07-22

**Authors:** Besnier Marc, Christophe Bonnet, Anne Lore Coudert

**Affiliations:** University of Poitiers, 86000 Poitiers, France

**Keywords:** Artificial intelligence, Primary care, History, Inteligencia artificial, Atención primaria, Historia

## Abstract

**Objective:**

Automating administrative tasks, such as compiling a patient's medical history, could help general practitioners in their daily work. AI performance has improved in recent decades, but skepticism among professionals limits its use in medical practice, due to fears of gaps and biases. This study attempts to evaluate the effectiveness of AI in recording patient histories compared to general practitioners.

**Design:**

Cross-sectional study.

**Site:**

Online study in France.

**Participants:**

French general practitioners recruited online.

**Intervention:**

We compared the performance of a general practitioner with that of AI in collecting a patient's history during an initial consultation.

**Main measurements:**

Medical histories were classified into categories: long-term chronic diseases, medical, surgical, obstetric, occupational, drug allergies, other allergies, and family history of cancer.

**Results:**

With 204 general practitioners, we obtained 942 patient histories. AI was more effective than traditional physicians in collecting patient histories, especially in older age groups and in categories such as allergies and family history. The relevance and reliability of those histories depend on the age and type of history reported. Older patients, obstetric history, and drug allergies were the most relevant factors.

**Conclusion:**

AI is expected to become a major player in the healthcare sector due to its efficiency and accuracy. However, there is still debate about its potential to replace doctors in tasks such as diagnosis, prevention, therapy, or patient information. The ethics of data collection and AI remain a subject of debate, with the importance of training healthcare professionals to ensure they respect medical and patient ethics. The study highlights the importance of integrating new technologies into general medicine and the doctor–patient relationship in the profession.

## Abstract (es)

**Objetivo:**

La automatización de tareas administrativas, como la recopilación del historial médico de un paciente, podría ayudar a los médicos generales en su trabajo diario. El rendimiento de la IA ha mejorado en las últimas décadas, pero el escepticismo de los profesionales limita su uso en la práctica médica, por temor a que haya lagunas y sesgos. Este estudio intenta evaluar la eficacia de la IA en el registro de los historiales de los pacientes en comparación con los médicos generales.

**Diseño:**

Estudio transversal.

**Emplazamiento:**

Estudio en línea.

**Participantes:**

Médicos generales franceses reclutados en línea.

**Intervención:**

Comparamos el rendimiento de un médico general con el de una IA en la recopilación del historial de un paciente durante una consulta inicial.

**Mediciones principales:**

Los historiales médicos se clasificaron en categorías: enfermedades crónicas de larga duración, médicas, quirúrgicas, obstétricas, laborales, alergias a medicamentos, otras alergias y antecedentes familiares de cáncer.

**Resultados:**

Con 204 médicos generales, obtuvimos 942 historiales de pacientes. La IA fue más eficaz que los médicos tradicionales en la recopilación de historiales de pacientes, especialmente en los grupos de edad más avanzada y en categorías como las alergias y los antecedentes familiares. La relevancia y la fiabilidad de esos antecedentes dependen de la antigüedad y del tipo de antecedentes que se indiquen. Los pacientes de edad avanzada, los antecedentes obstétricos y las alergias a medicamentos fueron los factores más relevantes.

**Conclusión:**

Se espera que la IA se convierta en un actor importante en el sector sanitario debido a su eficiencia y precisión. Sin embargo, sigue habiendo debates sobre su potencial para sustituir a los médicos en tareas como el diagnóstico, la prevención, la terapia o la información al paciente. La ética de la recopilación de datos y la IA siguen siendo objeto de debate, con la importancia de formar a los profesionales sanitarios para garantizar que respeten la ética médica y la del paciente. El estudio destaca la importancia de integrar las nuevas tecnologías en la medicina general y la relación médico/paciente en la profesión.

## Introduction

The shortage of physicians is affecting many countries, creating a need to reinvent healthcare.^1^ The global aging of the population, improvements in the monitoring of complex pathologies, and the increase in chronic pathologies are putting pressure on healthcare systems that are already under strain.^2^ The computerization of doctors’ offices has led to better traceability of patient data and more complete patient records.^3^ The use of new technologies has changed the daily grind of physicians. These tools enable better organization, save time, and improve the quality of life for physicians. But they can only be used if they meet certain criteria, including reliability and ease of use.^4^ The use of these tools must meet the criteria expected by users.^5^ Data security is a necessity and remains a priority.^6^ However, if these tools do not provide real value to practitioners, they will be neglected.^7^ Artificial intelligence (AI) is a recent concept, dating back to the 1980s. It has taken off in recent decades, thanks to deep learning and the new capabilities of computer hardware.^8^ However, until recently, its use was restricted to the healthcare sector.^9^ The hope of improved diagnostic and therapeutic capabilities has led to greater appropriation of these tools.^10^ As an example, physical AI are commonly use in surgery.^11^ Practitioners’ scepticism limits the use of these tools in medical practice.^12^ The current state of these tools makes practitioners fear gaps and biases in their use.^13^ However, users remain confident that artificial intelligence will be a key element in the years to come.^14^

Documentation burden is a major contributor to burnout in primary care and impacts physician well-being and workforce sustainability.^15^ The automation of administrative tasks would help GPs in their day-to-day work.^16^ Initial consultations can be long and strenuous. For the ordinary doctor the taking of a medical patient history is and has been one of the fundamental procedures.^17^ Most diagnoses can be made from the medical history.^18^ This result can be obtained if the patient's medical history is carefully taken and recorded in the medical file.^19^ However, taking a history is particularly laborious, making it difficult for practitioners to focus on the patient in the same time.^20^ This has an impact on the patient relationship and satisfaction expressed by patients.^21^ Even when patients fill in their own histories, there are still major discrepancies that can influence their management.^22^ This is sometimes even more complex in certain pathologies, such as cancer.^23^ Taking a medical history, particularly a family history, is an essential part of patient management.^24^ This helps us to better understand their specific risks, and to guide their treatment.^25^ A number of studies have shown that we find it difficult to fill in this history correctly, and that outside help could be beneficial.^26^ The evolution of artificial intelligence tools should make us question their current effectiveness in performing these tasks. Even if it cannot replace practitioners, artificial intelligence could be just as effective a helper in these supervised tasks.^27^

Our study aims to examine how well AI can gather patients’ medical histories. Our main outcome is the quantity of information about patients’ medical histories collected by clinicians and by AI during an initial consultation. We will also analyse the accuracy and reliability of this information, and of the tool itself. Our research hypothesis will be that AI collects more information than practitioners do on their own. We will examine the relevance of this information and its value to the medical record.

## Methods

### Study population

In a prospective, cross-sectional comparative study, we recruited general practitioners in private practice throughout France. Recruitment was done through professional associations and social networks. The selected physicians had to practice general medicine in private practice. Recruitment took place between January and March 2024. We expected the participation of two hundred general practitioners for a total of 500 patient files in order to obtain sufficient power for our study.^28^

### Study design

To take part in the study, we asked practitioners to fill in the histories of newcomer patients. Newcomers were defined as any patient seeing a practitioner for the first time, with no prior medical records at the practice. The visit had to be for care leading to ongoing follow-up, outside of an emergency setting. The inclusion criteria were any patient, regardless of age, who wished to be followed by the practitioner. Children were represented by their parents. Exclusion criteria included cognitive impairments, the inability to express non-consent, and refusal to participate in the study. All patients were informed about the use of the tool and the recording process and could withdraw their consent at any time. During each consultation, physicians conducted a standard clinical interview while simultaneously using an AI transcription tool operating in the background. Two independent medical histories were thus generated: one recorded manually by the physician and one automatically transcribed by the AI. Each patient was thus interviewed by a practitioner, with the doctor taking notes and the AI listening in the background. To facilitate the study and its reproducibility, we asked all practitioners to use the online version of the Nabla software (https://www.nabla.com/). This software combines an advanced ambient AI, dictation and real-time intelligence – seamlessly integrated to transcribe the content of a medical consultation in real time. As Nabla was available free of charge for a certain number of trials, no funding was required to complete the study. Each practitioner could include as many patients as he or she wished. The study was conducted from April to September 2024. All invited primary care physicians were assured that their identities would not be disclosed to the investigators, and participants gave informed consent before participating in the survey. Ethical approval for the study was obtained from the local ethic committee of the University of Poitiers.

Next, we provided the practitioners with feedback based on their data. We asked them to comment on these results according to several criteria. Firstly, we asked them to comment on the clinical relevance of the additional medical history recorded by the AI. We defined relevance to the medical record as data that enables better follow-up, more effective tracking of necessary screenings or identification of factors requiring monitoring. Reliability was defined as whether the recorded medical history had actually been reported by the patients and corresponded to their health issues. We also asked what percentage of the medical history the practitioners had retained in their records. Finally, we asked if they would recommend using AI for each type of medical history and patient population. These criteria were rated either on a Likert scale (0–10) or as a percentage of the data.

### Data collection

We used an anonymous patient history form. Each practitioner could log in at any time and submit his or her data and the AI's data simultaneously. There were no traceability of the data and no way to identify the practitioner or the place where the information was collected. For each file, the patient's age and sex were requested. These data were extracted. Two independent investigators conducted a blinded comparison of the medical histories taken by the physician and the AI and classified them into several broad history categories: chronic long-term illness, medical, surgical, obstetrical, occupational, drug allergy, other allergies, family history of cancer, other family history. This classification was designed to align with the categories used in the medical software commonly employed by practitioners. As a result, each category corresponded to a predefined field in most software programs, thereby facilitating data processing. In the event of a disagreement between the two investigators, arbitration was conducted by a third party.

### Data management and analysis

All correlation analyses were conducted using Spearman's rho; all group comparisons were conducted using the Mann–Whitney *U* test. These analyses were chosen because, unlike Pearson's *r* and *t*-tests, the dependent variable does not need to be continuous, and because most dependent variables in our analyses were ordinal outcomes. Alpha was set at 0.05 (two-tailed) for all analyses. All analyses were conducted using SPSS version 24.

## Results

A total of 204 GPs participated, for a total of 942 patient files. The distribution of patient files by age category is shown in Table 1. The patient group was homogeneous, with no significant differences between men and women in every age group (*p* = 0.38). We obtained a total of 75 infants under 6 yo (8%), 60 between 7 and 12 yo (6%), 25 between 13 and 18 yo (3%), 184 adults between 19 and 35 yo (20%), 313 between 36 and 65 yo (33%) and 285 aged 66 or more (30%).

**Table 1:** Patient distribution by age group and gender.

Patients age	Male	Female	Total
0–6	34	41	75
7–12	31	29	60
13–18	14	11	25
19–35	85	99	184
35–65	141	172	313
65+	151	134	285

Total	456	486	942

The distribution of events, by number of events, between general practitioners (GPs) and artificial intelligence (AI) is reported in Table 2.

**Table 2:** Distribution of the number of procedures by history category, age group and whether referred by general practitioners (GPs) or artificial intelligence (AI).

	Patients age						Total
	0–6	7–12	13–18	19–35	35–65	65+	
*Chronic long-term illness*							
GP	4	2	0	5	19	78	108
IA	7	3	1	8	42	121	182
P	0.34	0.65	0.32	0.4	<0.05	<0.05	<0.05

*Medical*							
GP	31	36	5	121	602	912	1707
IA	78	59	14	251	851	1231	2484
P	<0.05	<0.05	0.16	<0.05	<0.05	<0.05	<0.05

*Surgical*							
GP	4	6	1	24	131	302	468
IA	27	14	2	41	261	701	1046
P	<0.05	0.94	0.57	<0.05	<0.05	<0.05	<0.05

*Obstetrical*							
GP	0	0	0	12	31	71	114
IA	0	0	1	54	121	283	459
P	1	1	0.32	<0.05	<0.05	<0.05	<0.05

*Drug allergy*							
GP	1	2	1	11	27	26	68
IA	1	4	1	12	34	31	83
P	1	0.4	1	0.83	0.34	0.48	0.2

*Other allergies*							
GP	7	7	0	9	12	23	58
IA	41	67	3	32	64	27	234

*Family history of cancer*							
P	<0.05	<0.05	0.08	<0.05	<0.05	0.55	<0.05
GP	0	0	0	3	52	42	97
IA	0	0	1	31	154	131	317
P	1	1	0.32	<0.05	<0.05	<0.05	<0.05

*Other family history*							
GP	1	2	6	41	132	141	323
IA	51	42	22	79	617	872	1683
P	<0.05	<0.05	<0.05	<0.05	<0.05	<0.05	<0.05

With regard to long-term illness, AI was found to find more histories than practitioners when treating adults over 35 years of age (*p* < 0.05). It systematically identified more medical histories (*p* < 0.05). It also identified more surgical histories in young children under six and in all adults over 19 years of age. AI also found more obstetric and family cancer histories in adults (*p* < 0.05). However, it did not find more medication histories, but it did find more allergy histories in all age groups (*p* < 0.05). The same was true for other family histories, which were found more often by the AI (*p* < 0.05).

The secondary analysis (Table 3) showed that clinical relevance was higher for older populations and certain categories, such as obstetric history or drug allergies. The same was true for relevance to the clinical record. Reliability was excellent across all categories. Despite relevance sometimes being perceived as low, approximately 50% of the medical histories were retained by practitioners. The tool's recommendations varied depending on the relevance perceived by practitioners.

**Table 3:** Clinical relevance, relevance to the medical record, reliability of the history recorded by AI, percentage of retained histories find by AI and recommendation by practitioners.

	Patients age						Total
	0–6	7–12	13–18	19–35	35–65	65+	
*Chronic long-term illness*							
Clinical relevance	1.6	1.4	0	6.2	7.4	8.9	4.2
Relevance to medical record	2.1	2.2	0	6.4	8.5	9.2	4.8
Reliability (%)	87	95	94	92	90	92	92
Retained history (%)	16	14	0	64	86	92	45
Recommendation	2.1	3.1	0.8	7.4	8.1	9.7	5.2

*]Medical*							
Clinical relevance	4.1	3.1	4.3	2.1	3.2	2.1	3.1
relevance to medical record	3.8	3.4	4.5	2.3	4.1	2.8	3.5
Reliability	87	97	91	98	91	94	93
Retained history (%)	31	29	32	11	17	31	25
Recommendation	2.4	2.9	4.8	1.8	3.3	1.6	2.8

*Surgical*							
Clinical relevance	2.1	1.6	1.5	4.6	5.2	4.7	3.3
Relevance to medical record	3.8	2.2	1.5	5	6.8	7.3	4.4
Reliability	86	98	94	87	90	87	90
Retained history (%)	42	21	0	41	54	61	37
Recommendation	2.4	1.7	0.9	6.8	7.9	6.8	4.4

*Obstetrical*							
Clinical relevance	–	–	–	7.2	8.1	4.3	6.5
Relevance to medical record	–	–	–	7.4	7.9	8.1	7.8
Reliability	–	–	–	95	97	93	95
Retained history (%)	–	–	–	81	84	61	75
Recommendation	–	–	–	6.8	8.4	5.7	7

*Drug allergy*							
Clinical relevance	10	6	10	10	8.8	9.6	9
Relevance to medical record	10	6	10	10	9.2	8.8	9
Reliability	100	93	100	98	98	96	98
Retained history (%)	100	75	100	100	87	82	90
Recommendation	10	7.7	10	10	8.8	7.8	9

*Other allergies*							
Clinical relevance	3.1	2.8	0	4.1	4.7	5.3	3.3
Relevance to medical record	4.8	2.5	2.2	4.6	5.4	6	4.3
Reliability	98	90	93	98	89	86	92
Retained history (%)	61	27	33	52	58	48	57
Recommendation	2.3	1.8	0.9	3.8	4.2	4.5	2.9

*Family history of cancer*							
Clinical relevance	–	–	–	1.7	3.2	2.9	2.6
Relevance to medical record	–	–	–	4.9	5.7	6.9	5.8
Reliability	–	–	–	91	86	97	91
Retained history (%)	–	–	–	38	49	67	51
Recommendation	–	–	–	3.6	4.9	6.8	5.1

*Other family history*							
Clinical relevance	1.1	1.8	1.4	2	2.6	2.5	1.9
Relevance to medical record	1.2	1.6	1.3	2.1	3.2	3.7	2.2
Reliability	89	91	85	90	79	81	86
Retained history (%)	4	8	8	12	21	16	11
Recommendation	0.8	1	0.7	1.1	2	2.1	1.3

*]Total*							
Clinical relevance	3.7	2.8	2.9	4.7	5.4	5	4
Relevance to medical record	4.3	3	3.3	5.3	6.3	6.6	4.8
Reliability	91	94	93	94	90	91	92
Retained history (%)	42	42	45	53	58	57	50
Recommendation	3.3	3	3	5	6	5.6	4.3

## Discussion

### Summary of major findings

Artificial intelligence is becoming part of our daily lives.^29^ Our study shows the positive impact this technology can have on the daily task of collecting patient histories. Our prospective study found that artificial intelligence was more effective than GPs at collecting data across a wide range of history categories and age groups. The impact on older age groups, who had the most antecedents, was significant, with a notably higher number of events involving artificial intelligence. The same was true for categories such as allergies and family history, for which practitioners provided significantly less information.

Practitioners had mixed feelings when it came to chronic long-term illnesses. While some seemed worth adding, others had little impact on patient care. However, the majority acknowledged the value of administrative reporting when they were declared. This could encourage reporting of comorbidities, such as heart failure following a myocardial infarction.

The medical history reported by the AI was often deemed unnecessary by practitioners. This was due to a lack of added value in the patient's record (e.g. alopecia), long-term clinical relevance (e.g. cough), precision regarding the aetiology (e.g. dizziness) or temporal context for the event (e.g. ankle sprain). Practitioners found it time-consuming to sift through this information to retain only the most relevant details, and for this reason would not recommend using AI.

The surgical history reported by the AI was classified into two categories. The first category was of no interest to clinicians due to its minimal long-term impact, such as ingrown toenails, or the lack of need for follow-up, such as the removal of benign skin lesions. Conversely, several items warranted a mention in the records, such as surgeries with the potential for late complications (e.g. sigmoidectomy), conditions requiring urgent exclusion (e.g. appendicitis) and the need for long-term follow-up (e.g. complex skin lesion).

Obstetric history after the age of 18 was frequently cited by practitioners, who acknowledged that they had not reported it. They were particularly interested in certain cases, especially those involving HELLP syndrome, impact on future pregnancies (cesarean section) and the need for follow-up (gestational diabetes).

Practitioners agreed on drug allergies, which often involved related molecules. However, they did not see the value in documenting common allergies, whether food-related or environmental. Indeed, there was a concern that these issues might not be documented, as they were likely to be cases of intolerance rather than allergy. Furthermore, they did not see the clinical value in doing so. Some practitioners specified that if a serious event had occurred, it would already be noted in the medical history (e.g. angioedema).

Family history was generally considered relevant. A history of cancer was considered relevant, with a focus on monitoring and recommending targeted screening for colorectal cancer. However, doubts persisted regarding the accuracy and exact aetiology of other medical histories, raising concerns about unnecessary heightened vigilance. This could prompt practitioners to re-interview patients to better understand these details and refine individualised monitoring strategies.

So, even though the AI identified a larger number of prior conditions, these were not always relevant and could clutter up medical records rather than help practitioners. Certain age groups and categories of medical history appear to be eligible for this assistance, but healthcare providers must remain vigilant to verify the accuracy of the information identified by the AI.

### Are GPs in competition with AI?

Ambient AI scribes have emerged as a possible solution to reduce the documentation burden. Ambient voice recognition draft clinical notes and may allow physicians to focus more fully on patient care.^30^, ^31^ Early studies show improvement in well-being and documentation efficiency, including reduced documentation time and higher clinician satisfaction.^32^, ^33^ Many experts believe that AI is poised to become a major player in the healthcare sector thanks to its greater efficiency and precision.^34^, ^35^ However, debates are still underway as to whether AI can become powerful enough to replace doctors in tasks such as diagnosis, prevention,^36^ and therapy^37^, ^38^ or patients information's.^39^ There are still doubts, particularly concerning artificial intelligence's emotional and empathic capacities,^40^ as well as its ability to treat medicine as an art.^41^ Talking to patients and gathering their stories is part of this, to create a special bond and facilitate the therapeutic alliance.^17^, ^42^ These criteria are still essential if practitioners are to use AI.^43^ The latter express doubts as to whether the tool can replace them in these tasks.^14^

However, when it comes to collecting and classifying data, its effectiveness is much more widely appreciated. Complex tasks can be laborious for practitioners, and they are confident that artificial intelligence can handle them.^44^

Compared to other studies, we find that AI-powered scribe systems show definite potential for collecting patient information.^45^ It appears that these findings add to the growing body of research demonstrating the value of these tools.^30^, ^46^ There remains a debate about the ethics of data collection and AI.^47^ It seems just as important to train healthcare professionals as it is to continue ensuring that the development of these tools respects the patient and the rules of medical ethics.^48^ Ethics must remain at the heart of the debate if doctors are to retain confidence in this tool and patients are to tolerate its use in their care.^49^ The constant evolution of this tool and its use should make us both optimistic and cautious about its appropriation by tomorrow's doctors.^50^

### Strength and limitation

This study has several strengths. It is based on a prospective approach involving a large number of general practitioners, using a reproducible method applicable to real consultation conditions. Using the same artificial intelligence tool for data collection under real consultation conditions lends considerable strength to this study.

However, this study also has certain limitations. Data collection was carried out without any external control, including systematic verification by a third party. There is a selection bias among the participants. The physicians who agreed to participate in the study were volunteers and were likely already interested in AI. Patients who agreed to participate in the study also represent a selection bias The practitioners knew that they were being compared to an AI system resulting in an observational bias. This bias suggests that their performance may have differed from what they would usually produce. However, we can also assume that they may have performed better than usual, thereby increasing the reliability of our results. To end with, there is a lack of standardization regarding the number of patients per doctor.

## Conclusion

This study is part of a broader reflection of integrating new technologies into general medicine. In the context of a shortage of GPs, the use of AI to automate time-consuming tasks for doctors, such as collecting administrative or medical data, is attracting growing interest. While tools like ambient AI scribe could be valuable aids, their effectiveness and respect for ethical principles in medical practice must be assessed. The results of this study encourage further research. It is important not to neglect the doctor-patient relationship at the heart of the general practice profession.

## Authors’ contributions

Besnier Marc carried out the preliminary study, the statistics and the first draft.

Christophe Bonnet and Anne Lore Coudert helped us to reflect on the concept of the study and to write the paper in its present form. They took part in data analysis.

## Ethical approval

Our study has received the approval of the Data Protection Officer from the university of Poitiers.

## Consent to participate

Every participant has given a written consent to their participation in our study.

## Consent to publish

Every author gave their consent to publish this article.

## Human ethics

Our study was approved by the ethics committee of the University of Poitiers.

## Clinical trial number

Not applicable.

## Funding

No financial support was needed.

## Declaration of competing interests

The authors report no conflict of interest.

## Data availability

All data from our study are available for future reference. The data from our study are available upon request for ethical reasons.
